# Association Between Diabetes and COVID-19: A Retrospective Observational Study With a Large Sample of 1,880 Cases in Leishenshan Hospital, Wuhan

**DOI:** 10.3389/fendo.2020.00478

**Published:** 2020-07-14

**Authors:** Zeming Liu, Jinpeng Li, Jianglong Huang, Liang Guo, Rongfen Gao, Kuan Luo, Guang Zeng, Tingbao Zhang, Meilin Yi, Yihui Huang, Jincao Chen, Yibin Yang, Xiaohui Wu

**Affiliations:** ^1^Department of Plastic Surgery, Zhongnan Hospital of Wuhan University, Wuhan, China; ^2^Department of Thyroid and Breast Surgery, Zhongnan Hospital of Wuhan University, Wuhan, China; ^3^Department of Rheumatology and Immunology, Tongji Medical College, Tongji Hospital, Huazhong University of Science and Technology, Wuhan, China; ^4^Department of Neurosurgery, Wuhan Puren Hospital, Wuhan, China; ^5^Department of Urology, Zhongnan Hospital of Wuhan University, Wuhan, China; ^6^Department of Neurosurgery, Zhongnan Hospital of Wuhan University, Wuhan, China; ^7^Department of Burn and Plastic Surgery, College of Traditional Chinese Medicine, Three Gorges University, Yichang Hospital of Traditional Chinese Medicine, Yichang, China; ^8^Department of Respiratory and Critical Care Medicine, Zhongnan Hospital of Wuhan University, Wuhan, China

**Keywords:** clinical courses, comorbidity, Coronavirus disease 2019 (COVID-19), diabetes mellitus, prognosis

## Abstract

**Aims:** This study aimed to investigate the clinical courses and outcomes of diabetes mellitus patients with coronavirus disease 2019 (COVID-19) in Wuhan.

**Methods:** This study enrolled 1,880 consecutive patients with confirmed COVID-19 in Leishenshan Hospital. We collected and analyzed their data, including demographic data, history of comorbidity, clinical symptoms, laboratory tests, chest computed tomography (CT) images, treatment options, and survival.

**Results:** The percentages of patients with diabetes among the severe and critical COVID-19 cases were higher than those among the mild or general cases (89.2%, 10.8 vs. 0%, *p* = 0.001). However, patients with and without diabetes showed no difference in the follow-up period (*p* = 0.993). The mortality rate in patients with or without diabetes was 2.9% (*n* = 4) and 1.1% (*n* = 9), respectively (*p* = 0.114). Univariate and multivariate Cox regression analyses and the Kaplan-Meier curves did not show any statistically significant differences between patients with and without diabetes (all *p* > 0.05).

**Conclusions:** Our study results suggested that diabetes had no effect on the prognosis of COVID-19 patients but had a negative association with their clinical courses. These results may be useful for clinicians in the management of diabetic patients with COVID-19.

## Introduction

Coronavirus disease 2019 (COVID-19) cause by severe acute respiratory syndrome–coronavirus 2 (SARS-CoV-2) was first identified in Wuhan, China, and has subsequently and quickly spread to more than 70 countries, including the United States, Spain, and Italy (1, 2). SARS-CoV-2 could cause severe, even lethal pneumonia and lung failure. As of April 25, 2020, more than 2,800,000 confirmed cases and 190,000 deaths of COVID-19 have been reported worldwide (3).

As an emergency specialty field hospital hosted by the Zhongnan hospital, Leishenshan Hospital was put into use in February to provide treatment for COVID-19 patients in Wuhan, marking a milestone in China's battle against COVID-19. In Leishenshan Hospital, 1,880 patients were diagnosed with COVID-19 between February 8, 2020 and April 15, 2020. Among those patients, 139 cases had a previous diagnosis of diabetes mellitus.

Diabetes mellitus (DM) is a common endocrine disease (4) and widely known as a chronic, low-grade inflammatory disease caused by long-term immune system imbalance, metabolic syndrome, or nutrient excess (5, 6). Since the outbreak of COVID-19, several studies have been conducted to examine the relationship between diabetes and COVID-19 (1, 7, 8). However, their sample sizes were relatively small, and they did not clarify whether diabetes was a predictor of poor clinical outcomes and higher mortality of COVID-19. In this study, we investigated the association between diabetes as a comorbidity and negative clinical courses and outcomes of COVID-19 in a large sample of patients from a single hospital in Wuhan, China. The findings are expected to inform follow-up clinical treatment for patients with both diseases and COVID-19.

## Materials and Methods

### Study Design and Participants

This single-center retrospective observational study was conducted on 1,880 patients diagnosed with COVID-19 at Leishenshan Hospital. After excluding cases without information about the history of diabetes, the final sample included 934 patients. Data were collected on admission or during hospitalization by attending physicians and documented in the form of electronic medical records. This study was approved by the Ethics Commission of the Zhongnan Hospital of Wuhan University (Approval number: 2020074) and carried out in accordance with the principles of the Declaration of Helsinki revised in 2008. The need for informed consent from patients was waived due to the time constraints during the COVID-19 emergency.

### Data Collection

We collected data of 1,880 patients from their medical records. These data included age, sex, comorbidities, levels of illness severity (mild, moderate, severe, and critical), signs and symptoms, treatment options (i.e., antiviral therapy, antibiotic therapy, traditional Chinese medicine therapy, anticoagulation therapy, and administration of corticosteroid and Vitamin C), laboratory findings, and computed tomography (CT) images. All the data were reviewed and analyzed by two senior physicians.

### Outcomes

The primary outcomes of this study included the survival of patients (alive vs. dead) and their highest level of illness severity. Another important primary outcome was the patients' CT score—a semi-quantitative scoring system—generated according to the features of CT images. Each of the CT image features, namely ground-glass opacities, reticulation or cords change, consolidation, and pleural effusions, was assigned 1 point. Score 1 was the sum of points, and score 2 ranged from 0 to 4 points based on the area of lung involvement: no involvement, 0; <25% involvement, 1; 26–50% involvement, 2; 51–75% involvement, 3; and 76–100% involvement, 4. The total score was the sum of score 1 and score 2.

### Statistical Analysis

Normally distributed continuous variables are expressed as means ± standard deviations, and non-normally distributed continuous variables as medians and interquartile ranges. Meanwhile, categorical variables are described as frequencies and percentages. The independent *t*-test or Mann-Whitney *U*-test was conducted to compare continuous variables between the group of patients with diabetes (Group 2) and that of patients without diabetes (Group 1). Meanwhile, the χ^2^-test or Fisher's exact test was used to analyze the associations between categorical variables. Cox regression analysis and the Kaplan-Meier survival curves were performed to explore the prognosis of patients with and without diabetes. Besides, curve fitting analysis was conducted to further evaluate the association between the CT score and the duration from symptom onset (in days) in both groups. Two-sided *p* < 0·05 were considered as statistically significant, and all statistical analyses were performed using SPSS (version 23.0 for windows).

## Results

### Study Participants' Demographic and Clinical Information and Laboratory Findings

Table 1 shows that Group 2 consisted of 139 patients whereas Group 1 had 795 patients. The two groups showed no differences in gender distribution (*p* = 0.076), means of age (*p* = 0.773), and the number of patients with malignancy, nervous system disease, and digestive system disease (all *p* > 0.05). However, they showed a significant difference in the number of patients with cardiovascular or pulmonary disease (both *p* < 0.05). The percentages of patients with diabetes among the severe and critical COVID-19 cases were higher than among the mild or general cases (89.2%, 10.8 vs. 0%, *p* = 0.001). However, no difference was observed in the follow-up period between patients with and without diabetes (*p* = 0.993). The mortality rate was 2.9% (*n* = 4) in Group 2 and 1.1% (*n* = 9) in Group 1 (*p* = 0.114). As shown in Table 2, the positive rate of IgG against SARS-CoV-2 among patients with diabetes was lower than that among those without diabetes (82.5 vs. 91.8%, *p* = 0.029).

**Table 1:** Demographics and clinical characteristics of patients with COVID-19.

**Covariate**	Group 1^*^ (*n* = 795)	**Group 2^#^ (*n* = 139)**	***P*-value**
Age, year	61.6 ± 14.5	64.5 ± 10.0	0.076
Sex			
Male	388 (48.8%)	66 (47.5%)	0.773
Female	407 (51.2%)	73 (52.5%)	
Comorbidity			
Cardiovascular disease	275 (34.6%)	89 (64.0%)	<0.001
Pulmonary disease	90 (11.8%)	3 (2.4%)	0.001
Malignancy	52 (6.5%)	14 (10.1%)	0.134
Nervous system disease	47 (5.9%)	12 (8.6%)	0.224
Digestive system disease	39 (4.9%)	7 (5.0%)	0.948
The highest level of severity			
Mild and general	19 (2.4%)	0 (0)	0.001
Severe	739 (93.3%)	124 (89.2%)	
Critical	34 (4.3%)	15 (10.8%)	
Status of illness when admission			
Mild	286 (38.2%)	43 (30.9%)	0.150
General	211 (28.2%)	37 (26.6%)	
Severe	235 (31.4%)	53 (38.1%)	
Critical	17 (2.3%)	6 (4.3%)	
Symptoms when admitted to the hospital			
Fever or Myalgia	559 (78.8%)	95 (76.6%)	0.577
Respiratory system symptoms	571 (80.5%)	104 (83.9%)	0.382
Digestive system symptoms	77 (10.9%)	13 (10.5%)	0.901
Nervous system symptoms	22 (3.1%)	5 (4.0%)	0.590
Antiviral therapy	449 (98.0%)	86 (97.7%)	0.693
Antibiotic therapy	297 (98.3%)	47 (97.9%)	0.590
The appliance of Vitamin C	106 (98.1%)	19 (100.0%)	1.000
Traditional Chinese medicine therapy	687 (99.7%)	118 (100.0%)	1.000
Anticoagulation treatment	98 (12.3%)	21 (15.1%)	0.364
Use of corticosteroid	82 (10.3%)	16 (11.5%)	0.671
Deaths	9 (1.1%)	4 (2.9%)	0.114
Follow-up days	22.4 ± 9.7	22.4 ± 9.3	0.993

* *Group 1 for patients without diabetes*.

# *Group 2 for patients with diabetes*.

**Table 2:** Laboratory results in the COVID-19 patients with or without diabetes.

**Covariate**	**Group 1 (*n* = 795)**	**Group 2 (*n* = 139)**	***P*-value**	**Reference**
Interleukin-6, pg/mL	2.16 (1.50–7.34)	3.69 (1.50–7.47)	0.133	0–7
Procalcitonin, ng/mL	0.04 (0.03–0.07)	0.05 (0.03–0.08)	0.142	<0.05
Alanine aminotransferase, U/L	23.00 (14.83–39.00)	21.00 (13.00–34.50)	0.054	9–50
Aspartate aminotransferase, U/L	21.00 (16.00–29.00)	18.00 (14.50–25.00)	<0.001	15–40
Albumin, g/L	35.95 (33.30–38.50)	35.90 (33.10–38,70)	0.880	40–55
Creatine kinase, U/L	47.00 (33.00–71.25)	49.00 (30.00–74.00)	0.817	18.0–198
Lactate dehydrogenase, U/L	197.50 (167.00–234.25)	194.00 (172.00–231.00)	0.941	125–343
Total bilirubin, μmol/L	9.10 (6.68–12.00)	9.20 (6.75–12.45)	0.420	5–21
Creatinine, μmol/L	65.90 (55.40–77.16)	66.00 (54.70–83.55)	0.545	64–104
BUN, mmol/L	4.90 (3.90–6.00)	5.20 (4.30–6.60)	0.004	2.8–7.6
Prothrombin time, s	11.40 (11.00–11.90)	11.40 (10.90–12.00)	0.784	9.4–12.5
Activated partial thromboplastin time, s	27.00 (24.20–30.40)	27.7 (24.98–30.48)	0.311	25.1–36.5
Fibrinogen, g/L	3.24 (2.58–3.99)	3.37 (2.65–4.08)	0.170	2.0–4.0
D-dimer, ng/mL	0.59 (0.27–1.42)	0.76 (0.32–1.91)	0.030	<0.50
White blood cell count, × 10^9^/*L*	5.73 (4.67–7.07)	5.68 (4.77–7.15)	0.785	3.5–9.5
Neutrophil count, × 10^9^/*L*	3.44 (2.51–4.54)	3.60 (2.51–4.86)	0.433	1.8–6.3
Lymphocyte count, × 10^9^/*L*	1.45 (1.11–1.84)	1.46 (1.10–1.81)	0.785	1.1–3.2
Monocyte count, × 10^9^/*L*	0.52 (0.41–0.67)	0.49 (0.41–0.62)	0.149	0.1–0.6
Red blood cell count, × 10^9^/*L*	4.01 (3.60–4.37)	3.94 (3.49–4.30)	0.259	4.3–5.8
Hemoglobin, g/L	122.00 (111.00–133.00)	121.00 (108.00–132.00)	0.215	130–175
Platelet count, × 10^9^/*L*	231.00 (183.00–287.75)	205.00 (163.00–259.00)	0.004	125–350
IgM (+) of SARS-CoV-2, No. (%)	101 (38.1%)	19 (29.7%)	0.209	(–)
IgG (+) of SARS-CoV-2, No. (%)	234 (91.8%)	52 (82.5%)	0.029	(–)

### Survival Analysis

**Figure 1:**
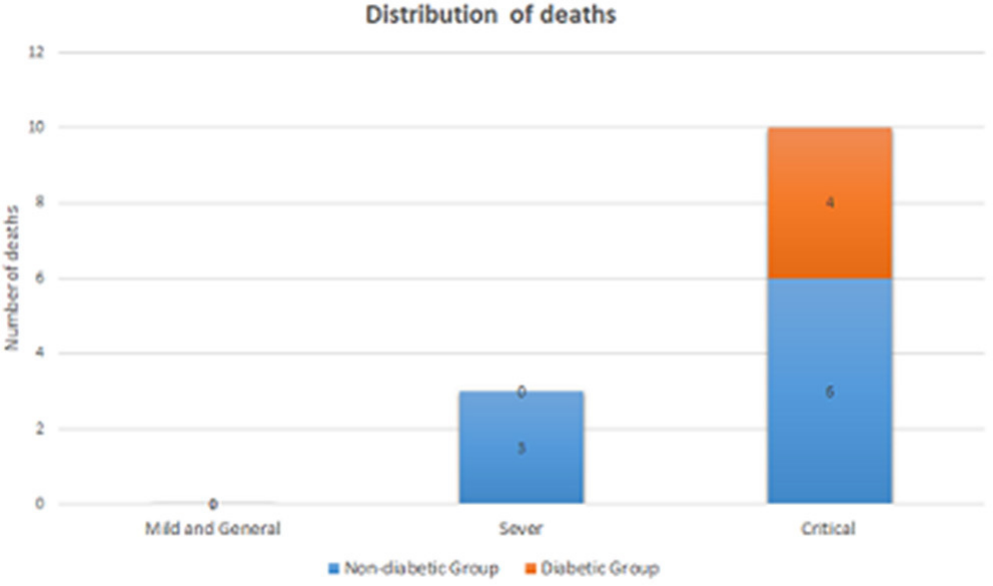
The distribution of the number of deaths by the level of illness severity in the DM group and the non-DM group.

Figure 1 presents the distribution of the number of deaths by the level of illness severity in both groups. There were six critical COVID-19 cases in Group 2, and three cases in Group 1. Besides, no deaths were observed among the severe cases in Group 2 while there were six deaths in Group 1. As shown in Table 3, the difference between the prognosis of Group 2 and that of Group 1 was demonstrated using either the univariate Cox regression analysis (*p* = 0.124) or the multivariate analysis (*p* = 0.256, Table 3). Similarly, the Kaplan-Meier curves showed no difference between the two groups (*p* = 0.111, in Supplemental Figure 1).

**Table 3:** Univariate and multivariate Cox regression analyses of diabetes for the mortality of patients with COVID-19.

**Covariate**	**Group**	**Cox regression analysis**			
		**HR**	**95 % CI**		***P*-value**
Univariate analysis	Group 1	ref			
	Group 2	2.522	0.777	8.191	0.124
Multivariate analysis^*^	Group 1	ref			
	Group 2	2.135	0.576	7.912	0.256

* *Adjust for age, the history of cardiovascular disease, D-dimer, WBC, and lymphocyte count*.

### Evaluation of Chest CT Images

**Figure 2:**
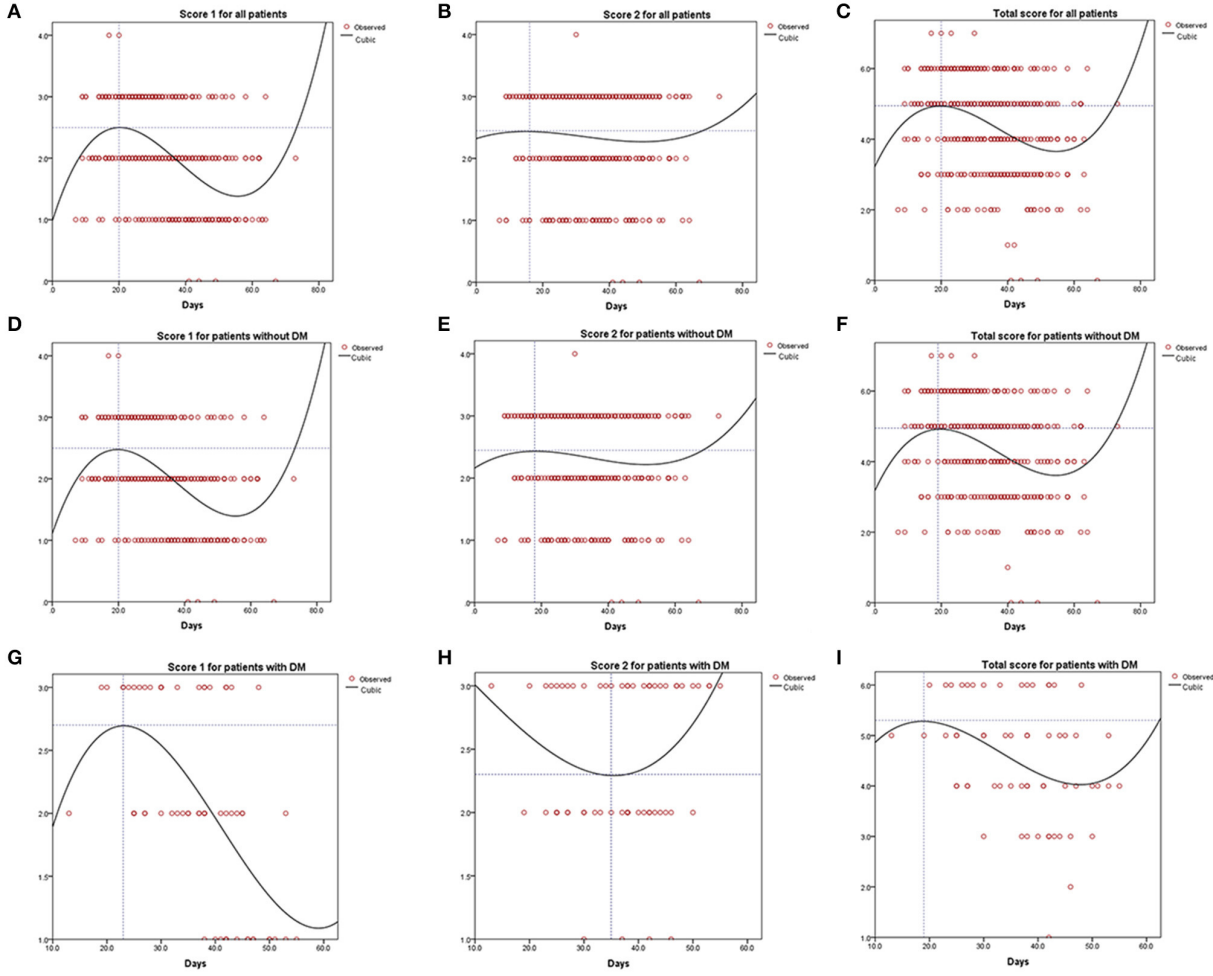
Curve fitting analysis for all the COVID-19 patients **(A–C)**, patients without diabetes mellitus **(D–F)**, and patients with diabetes mellitus **(G–I)**.

Score 1 for all patients and that for patients without diabetes present the same tendency, i.e., rising and then descending (Figures 2A–C for all patients vs. Figures 2D–F for patients without diabetes). Similarly, score 1 for patients with diabetes sharply increased and significantly decreased after peaking at 2.70 points on day 23 (Figure 2G). However, score 2 for patients with diabetes showed an inverse tendency (Figure 2H), reaching its nadir of 2.30 points on day 35. This inconsistent tendency might be because the largest area of lung involvement for patients with diabetes appeared earlier than that for all patients or those without diabetes. In patients with diabetes, the total score peaked at 5.30 points after 19 days (Figure 2I), exceeding the peak of 4.95 points on day 20 in all patients (Figure 2C) and that of 4.95 points on day 19 in patients without diabetes (Figure 2F). Curve fitting equation for Figure 2 and coefficients of each curve are shown in Supplemental Table 1.

## Discussion

The prevalence of diabetes mellitus is anticipated to increase substantially during the next decades worldwide and considered to be main cause of human deaths (9). People with diabetes are more susceptible to certain infectious diseases, such as staphylococcus aureus and mycobacterium tuberculosis, possibly because of their dysregulated immune system (10, 11). During the outbreak of SARS in 2003 in Guangzhou, Yang et al. reported that plasma glucose levels and diabetes were independent predictors for mortality and morbidity, and metabolic control might improve the prognosis in patients with SARS (12). Recently, COVID-19 has been a focal topic of research, and several investigations have focused on diabetes as a predictor of clinical course and prognosis of COVID-19 cases (13–16). Certain studies found that diabetes negatively affected medical complications, including mortality, in COVID-19 cases (13, 17, 18).

In our study, we collected data with a large sample from Leishenshan Hospital. We found that COVID-19 patients with DM were older and the proportion of patients with cardiovascular disease were higher among COVID-19 patients with DM than COVID-19 patients without DM. However, the latter had a higher proportion of pulmonary disease as comorbidities than the former. Furthermore, aspartate aminotransferase (AST) and platelet counts were lower in COVID-19 patients with diabetes than those without disease. In addition, non-diabetes-related comorbidities did not ameliorate the severity of illness on admission or improve survival outcomes, compared to diabetes-related ones. However, severe or critical COVID-19 cases were more prevalent among patients with diabetes than those did not suffer from this disease. A systematic review by Huang et al. revealed that diabetes was associated with mortality, severity, acute respiratory distress syndrome, and disease progression in patients with COVID-19 (19). The results of our present study differ from those of previous ones possibly because Leishenshan Hospital is a designated hospital for COVID-19, and the standard of care for patients with diabetes has become better. Further researches are needed in the future to generate more precise results.

Having assessed the association between diabetes with the severity of COVID-19, Wu et al. found out the proportion of diabetes as a comorbidity among severe COVID-19 cases was significantly higher than that among mild cases (14). Similarly, our data supported that the proportion of severe or critical COVID-19 cases among patients with diabetes was higher than that among those without diabetes. This means the clinical course of COVID-19 in patients with diabetes may be more severe than that in those without diabetes. Currently, the mechanisms behind this phenomenon remain unknown; however, high glucose levels may play a certain role in the impaired antibacterial neutrophil function and complications caused by chronic diabetes (9). In addition, the comorbidity with cardiovascular diseases, such as ischemic heart disease and heart failure, was reported to have an association with higher mortality due to COVID-19 (20). In our study population, 64.0% of patients with diabetes suffered from cardiovascular diseases, higher than that of those without diabetes. This may partly explain that the group of patients with diabetes had a higher proportion of COVID-19 cases in severe or critical conditions than the group of those without diabetes.

In our present study, biochemical laboratory test results showed that the increased AST level, an indicator of liver injury, was not observed in the blood of all patients, regardless of their status of diabetes. Furthermore, the two groups showed no difference in the levels of albumin and hemoglobin, suggesting that both groups had similar nutritional status. Having analyzed 138 COVID-19 patients, Wang et al. found that cytokine storms, sustained inflammatory response, and acute kidney injury might be associated with the mortality of COVID-19 patients (21). Cytokine storms also proved to be the main cause of eventual deaths among many patients with Ebola virus infections (13). In our study, however, patients with diabetes shared similar lymphocyte and neutrophil counts than patients without diabetes, the inflammation-related biomarkers (e.g., IL-6). These results indicate that the inflammatory response of patients with diabetes was not different from that of those without diabetes, resulting in similar rates of mortality due to COVID-19.

The use of angiotensin-converting enzyme 2 (ACE2) inhibitors and Angiotensin II Receptor Antagonists (ARBs) for COVID-19 patients has been of great controversy. ACE2 is the surface receptor for SARS-CoV-2 that directly interacts with the spike glycoprotein (22). Wrapp et al. has recently suggested that the affinity between ACE2 and the receptor binding domain of SARS-CoV-2 is much higher than that between ACE2 and the RBC of SARS-CoV (23). The expression of ACE2 in various organs, including the cardiovascular system, lungs, kidneys, and brain might explain why some COVID-19 patients died of multiple organ failure (24, 25). ACE inhibitors and ARBs may play a protective role in the treatment of COVID-19 cases (26). However, Cure et al. demonstrated that SARS-CoV-2 could enter cells by attaching to ACE2 enzymes and then cause infection. Furthermore, ACE inhibitors enhanced the sympathetic activity via the central stimulation and then increased pulmonary capillary leaking, possibly resulting in the development of ARDS. The authors suggested that the morbidity and mortality of COVID-19 among patients with diabetes would be higher if they used ACE inhibitors and ARBs (27). Our primary findings showed that as a comorbidity, diabetes did not increase the risk of mortality but negatively regulated the clinical course of COVID-19. Therefore, the treatment for COVID-19 patients should be appropriately adjusted.

Our study has serval limitations that need to be addressed. First, the retrospective, non-randomized nature led to sample heterogeneity. Second, we were not able to collect and analyze the characteristic data of patients with diabetes, such as the type of diabetes, glucose level, HbA1c and treatment options for diabetes due to the time limitation; hence, we could not analyze the data on anti-DM treatment although they could affect the clinical course and treatment outcome of COVID-19. Third, we did not examine the relative mechanism behind the effect of diabetes on COVID-19 in this study. Finally, the difference in disease progress and prognosis between the COVID-19 patients with or without diabetes may change with a longer follow-up period.

## Conclusion

Our findings which were contradictory to those of previous studies with large sample sizes, suggested that diabetes did not significantly impact the prognosis of COVID-19 patients but negatively affect their clinical course. This may be helpful for clinicians in managing COVID-19 patients with diabetes. However, future prospective studies with larger sample sizes should focus on examining whether patients with diabetes are more at risk of COVID-19.

## Data Availability Statement

The raw data supporting the conclusions of this article will be made available by the authors, without undue reservation, to any qualified researcher.

## Ethics Statement

The studies involving human participants were reviewed and approved by Ethics Commission of the Zhongnan Hospital of Wuhan University Zhongnan Hospital of Wuhan University. The Ethics Committee waived the requirement of written informed consent for participation.

## Author Contributions

ZL: conceptualization, writing—review, and editing. JH: formal analysis and writing—original draft preparation. JL, LG, and RG: investigation and data curation. KL and GZ: methodology and software. TZ, MY, and YH: software, visualization, and validation. JC, YY, and XW: supervision and project administration. All authors: contributed to the article and approved the submitted version.

## Conflict of Interest

The authors declare that the research was conducted in the absence of any commercial or financial relationships that could be construed as a potential conflict of interest.
