# Supplementary material for: Association Between Diabetes and COVID-19: A Retrospective Observational Study With a Large Sample of 1,880 Cases in Leishenshan Hospital, Wuhan
Source: Front Endocrinol (Lausanne). 2020 Jul 14;11:478. doi: 10.3389/fendo.2020.00478 (PMC7371935; doi:10.3389/fendo.2020.00478)

Supplemental Table 1.Curve fitting equations for Figure 2：

y=A+z(1)*x+z(2)*x^2^+z(3)*x^3^ , x= time in hospitalization

A, z(1), z(2), z(3) for each curve in Figure 2 are presented in the table below.

|  |  |  |  |  |
| --- | --- | --- | --- | --- |
| Figure 2 | A | z(1) | z(2) | z(3) |
| a | 0.98 | 0.17 | -0.01 | 5.05E-05 |
| b | 2.32 | 0.02 | 0.00 | 7.70E-06 |
| c | 3.22 | 0.20 | -0.01 | 6.09E-05 |
| d | 1.11 | 0.16 | -0.01 | 4.79E-05 |
| e | 2.16 | 0.03 | 0.00 | 1.20E-05 |
| f | 3.17 | 0.20 | -0.01 | 6.25E-05 |
| g | -0.16 | 0.28 | -0.01 | 6.94E-05 |
| h | 3.43 | -0.04 | 0.00 | 2.04E-05 |
| i | 3.01 | 0.28 | -0.01 | 0 |
|  |  |  |  |  |

Supplemental Figure 1. The Kaplan-Meier curves for the survival of COVID-19 patients with or without diabetes mellitus.


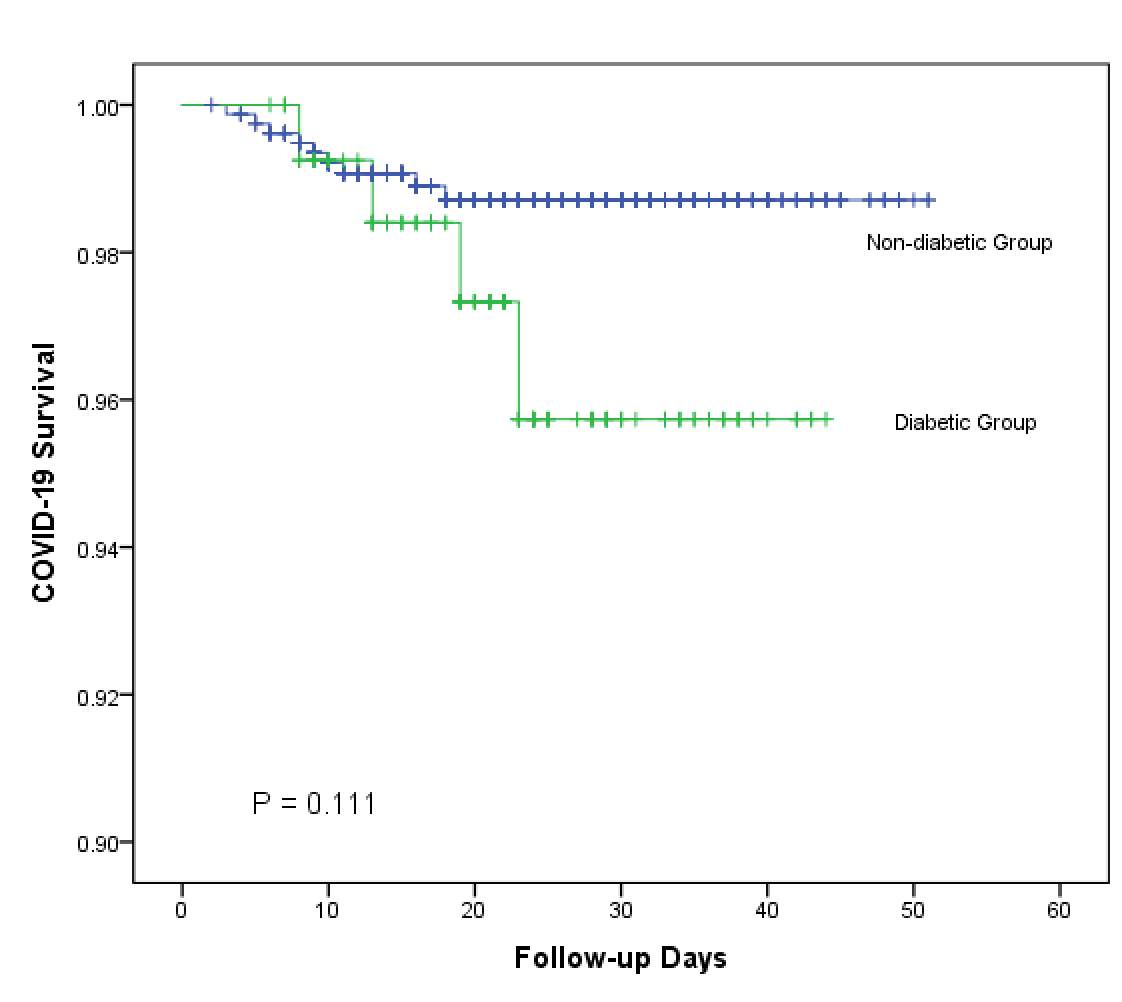

Supplement: Supplementary file 1 [file Data_Sheet_1.docx]
